# Myotube migration to cover and shape the testis of *Drosophila* depends on Heartless, Cadherin/Catenin, and myosin II

**DOI:** 10.1242/bio.025940

**Published:** 2017-11-09

**Authors:** Silke Rothenbusch-Fender, Katharina Fritzen, Maik C. Bischoff, Detlev Buttgereit, Susanne F. Oenel, Renate Renkawitz-Pohl

**Affiliations:** 1 Philipps-Universität Marburg, Fachbereich Biologie, Entwicklungsbiologie, Karl-von-Frisch Straße 8, 35043 Marburg, Germany; 2 DFG Research Training Group, Membrane Plasticity in Tissue Development and Remodeling, GRK 2213, Philipps-Universität Marburg, 35043 Marburg, Germany

**Keywords:** DWnt2, Thisbe, Stumps, FGF, Testes tubules, Muscles

## Abstract

During *Drosophila* metamorphosis, nascent testis myotubes migrate from the prospective seminal vesicle of the genital disc onto pupal testes and then further to cover the testes with multinucleated smooth-like muscles. Here we show that DWnt2 is likely required for determination of testis-relevant myoblasts on the genital disc. Knock down of fibroblast growth factor receptor (FGFR) *heartless* by RNAi and a dominant-negative version revealed multiple functions of Heartless, namely regulation of the amount of myoblasts on the genital disc, connection of seminal vesicles and testes, and migration of muscles along the testes. Live imaging indicated that the downstream effector Stumps is required for migration of testis myotubes on the testis towards the apical tip. After myoblast fusion, myosin II is needed for migration of nascent testis myotubes, in which Thisbe-dependent fibroblast growth factor (FGF) signaling is activated. Cadherin-N is essential for connecting these single myofibers and for creating a firm testis muscle sheath that shapes and stabilizes the testis tubule. Based on these results, we propose a model for the migration of testis myotubes in which nascent testis myotubes migrate as a collective onto and along the testis, dependent on FGF-regulated expression of myosin II.

## Abstract (precis)

**Summary:** *Drosophila* testes and mammalian seminiferous tubules are surrounded by a muscle layer. *Drosophila* myotubes migrate towards testes in dependence of the FGF receptor Heartless, myosin II and Cadherin-N.

## INTRODUCTION

Cell migration is essential for many developmental and physiological processes throughout the animal kingdom, and is also implicated in diseases, e.g. cancer metastasis (Roca-Cusachs et al., 2013). In *Drosophila*, various single as well as collective cell migration processes have been described, such as border cell migration in the ovary and embryonal mesoderm migration (Pocha and Montell, 2014).

In the current study, we focus on the migration of muscle cells during the development of the male reproductive tract of *Drosophila*. The five different organs of the inner male reproductive system of *Drosophila* develop from two different tissues. The testes are of gonadal origin located in segment A5, whereas the somatic parts arise from a single genital imaginal disc (hereafter called genital disc) in segments A8/A9/A10 (Estrada et al., 2003; Greig and Akam, 1995; Stern, 1941). During metamorphosis, the genital disc and pupal testes grow towards each other, and the developing seminal vesicles fuse with the terminal epithelium of the testes (Kozopas et al., 1998; Nanda et al., 2009; Stern, 1941). Nascent myotubes migrate over the developing seminal vesicles onto the pupal testes and build the muscle sheath surrounding the adult testis (Kozopas et al., 1998; Kuckwa et al., 2016). This musculature is composed of multinucleated, smooth-like myofibers (Susic-Jung et al., 2012).

**Fig. 1.**
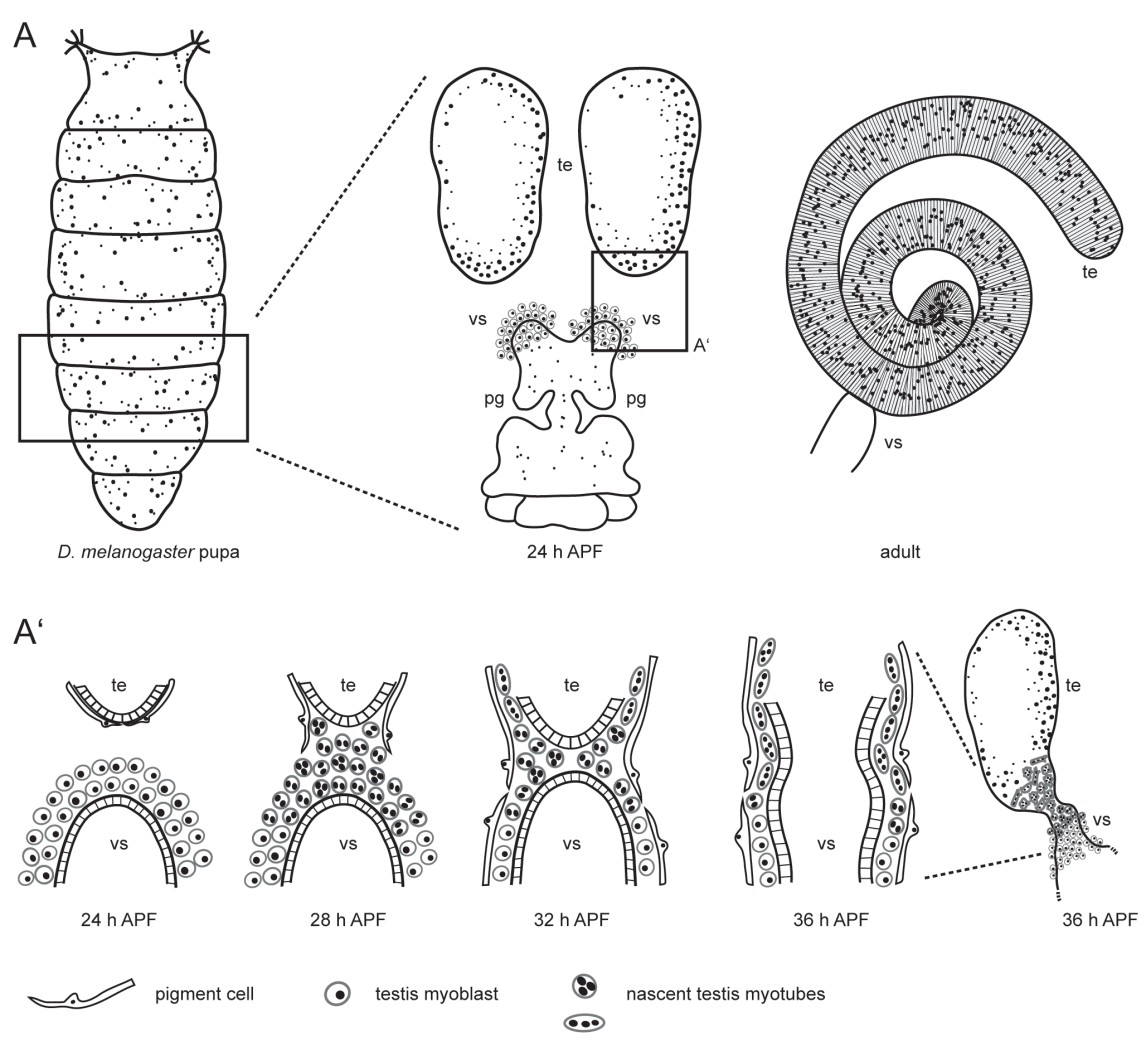
**Scheme of *Drosophila* testis myotube migration.** (A) The male reproductive tract develops during metamorphosis. At 24 h APF, the single genital disc and paired testes (te) are separate organs. The seminal vesicles (vs) and the paragonia (pg) already start to grow. In the adult, the tubular testis is connected to the seminal vesicle. (A’) During metamorphosis, the prospective seminal vesicles and testes grow towards each other and fuse. On genital discs 24 h APF, testis-relevant myoblasts accumulate on the prospective seminal vesicle. Pigment cells cover the pupal testis. At 28 h APF, myoblasts fuse to build multinucleated testis myotubes. These nascent testis myotubes migrate beneath the pigment cells onto the pupal testis, while pigment cells migrate from the testis onto the developing seminal vesicle. By 36 h APF, the epithelia of seminal vesicles and the terminal epithelium of the testes have fused. Modified after Bodenstein (1950), Kozopas et al. (1998), Kuckwa et al. (2016).

Myoblasts of the genital disc build muscle sheaths for all parts of the male reproductive system (Susic-Jung et al., 2012). The myoblasts that form the testis muscle sheath originate from a common pool and accumulate during the first day of metamorphosis on the prospective seminal vesicles of the genital disc (Fig. 1A). Founder-cell-like (FC-like) myoblasts and fusion-competent-myoblast-like (FCM-like) cells start to fuse around 28 h after puparium formation (APF) to build multinucleated myotubes (Kuckwa et al., 2016). Around 30 h APF, the multinucleated nascent myotubes begin to migrate from the genital disc towards the testis, contact the gonad at the distal end, and migrate further to cover the entire testis (Fig. 1A′) (Kozopas et al., 1998; Kuckwa et al., 2016). Migration of testis myotubes is independent of successful fusion of testis-relevant myoblasts (Kuckwa et al., 2016). Early evidence indicated that this migration process might be dependent on the presence of the Wnt ligand DWnt2 in addition to, or as a consequence of, the failure of pigment cell migration, since in DWnt2 mutant males smooth-like muscles do not accumulate on the testis (Kozopas et al., 1998).

Another relevant pathway for the development of the male reproductive organs of *Drosophila* is fibroblast growth factor (FGF) signaling. The FGF receptor (FGFR) Breathless (Btl) and its ligand Branchless recruit larval mesodermal cells, which become epithelial and give rise to paragonia and seminal vesicles (Ahmad and Baker, 2002). Btl is also essential for cell migration during embryonal tracheal development (Glazer and Shilo, 1991) and for directed cell migration of midline glial cells (Klämbt et al., 1992). The second FGFR in *Drosophila*, Heartless (Htl), becomes activated by its ligands Thisbe (Ths) and Pyramus (Pyr) (Gryzik and Müller, 2004; Stathopoulos et al., 2004) and is implicated in various migration processes. Htl is expressed in the mesoderm during gastrulation (Shishido et al., 1993), where it is necessary for the epithelial-mesenchymal transition, i.e. the dorsolateral migration of individual mesodermal cells along the ectoderm (Gisselbrecht et al., 1996). Htl is also needed during ovarian muscle tissue development (Irizarry and Stathopoulos, 2015). Htl-dependent FGF signaling also guides the migration of founder cells of the longitudinal midgut muscles during *Drosophila* embryogenesis (Kadam et al., 2012; Reim et al., 2012). During migration, these longitudinal founder cells fuse with fusion-competent myoblasts to build syncytia. Rudolf et al. (2014) have shown that this migration and fusion process is dependent on cytoskeletal rearrangements, particularly Arp2/3-induced actin polymerization. The function of cytoskeleton components and their regulators is also implicated in other cell migration processes. In vertebrate cells, Arp2/3 is needed for actin nucleation in lamellipodia-dependent cell migration (Campellone and Welch, 2010), while non-muscle myosin II plays a fundamental role in promoting directional cell migration (Vicente-Manzanares et al., 2009).

In *Drosophila*, cadherins such as Shotgun (Shg, the *Drosophila* homologue of E-Cadherin) and Cadherin-N (Cad-N) can mediate adhesion between neighboring cells. Catenins, such as beta-catenin, mediate the link between cadherins and the cytoskeleton (Bulgakova et al., 2012). Exemplarily, during the epithelial-mesenchymal transition in the *Drosophila* embryo, a series of changes in cell polarity, cell adhesion, and motility occur. Cells undergo a switch from an adhesive state towards a migrating state (Lim and Thiery, 2012). Thereby, the transcription factor Snail down-regulates epithelial genes, e.g. Shg, while Twist induces the transcription of Cad-N in the mesoderm (Leptin and Grunewald, 1990).

Here, we report first insights into the migration process of nascent myotubes from the prospective seminal vesicle onto the pupal testis and further towards the apical tip of the testis. Based on our results, we propose a model that links Ths- and Htl-dependent FGF signaling to myosin II-dependent processes during the migration of nascent myotubes from the genital disc onto the testes.

## RESULTS

### The adhesion molecule Cadherin-N is essential for building a continuous testis sheath

**Fig. 2.**
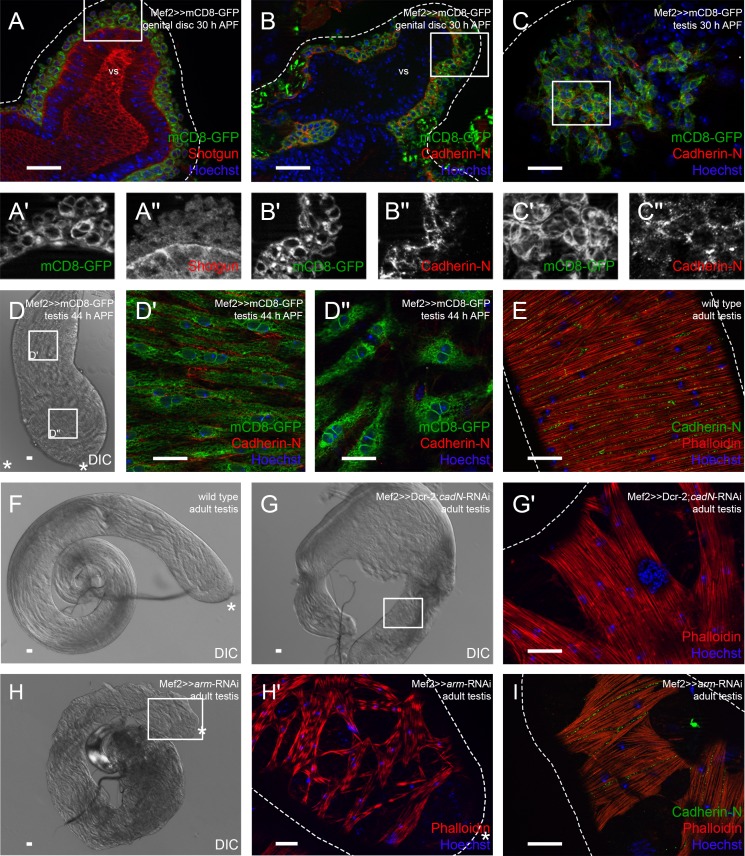
**Knock-down of *Cadherin-N* or *Armadillo* strongly reduces the adhesion between testis myotubes.** Immunofluorescence analyses of genital discs and testes. (A) Seminal vesicles 30 h APF stained or marked with anti-Shotgun (red), GFP (green; myoblasts and myotubes on genital discs and pupal testes marked with Mef2≫mCD8-GFP), and Hoechst (blue; nuclei). (A′,A″) Enlargement of boxed area in A, stained or marked as indicated. (B) Genital discs 30 h APF and (C) testis 30 h APF stained or marked with anti-Cad-N (red), GFP (green), and Hoechst (blue), magnification of prospective seminal vesicle is shown. (B′,B″,C′,C″) Enlargement of boxed area in B and C stained or marked as indicated. (D–D″) Testis 44 h APF. (D) Differential interference contrast (DIC) micrograph of testis 44 h APF, (D′,D″) enlargement of boxed area in D stained or marked with anti-Cad-N (red), GFP (green), and Hoechst (blue). (E) Adult testis stained with Hoechst (blue), Phalloidin to visualize F-actin (red), and anti-Cad-N (green). (F) DIC micrograph of wild-type testis. (G) DIC micrograph of *cad-N* knock-down testis; (G′) enlargement of boxed area in G showing Phalloidin (red) and Hoechst (blue) staining of testis muscle sheath. (H) DIC micrograph of *arm* knock-down testis; (H′) enlargement of boxed area in H showing Phalloidin (red) and Hoechst (blue) staining of testis muscle sheath. (I) Adult *arm* knock-down testis stained with Hoechst (blue), Phalloidin to visualize F-actin (red), and anti-Cad-N (green). Dotted lines reflect approximate shape of the organ. Asterisk, hub region; vs, seminal vesicle. Scale bars: 20 µm.

Since the *Drosophila* adult testis is encircled by a tight muscle sheath, we asked whether cadherins mediate the adhesion between myotubes in the testis muscle sheath before, during, and after migration. In expression analyses, we used Mef2-Gal4-driven UAS-mCD8-GFP flies to visualize myoblasts on genital discs and pupal testes at distinct time points. We observed that on genital discs 30 h APF, Shg (Cad-E) was expressed in the epithelium of the prospective seminal vesicle but was hardly detectable in myoblasts (Fig. 2A–A″).

By contrast, Cad-N was detected in the membranes of nascent myotubes on genital discs 30 h APF (Fig. 2B–B″) as well as on testes at 30 h APF (Fig. 2C–C″). Nascent myotubes of testes 44 h APF expressed Cad-N in correlation to their stage of migration. Specifically, myotubes at the basal testis end already started to elongate and to build a sheath, and expressed Cad-N (Fig. 2D,D′), whereas Cad-N was barely detected in nascent myotubes near the apical tip in mCD8-GFP-labeled myotubes in an optical section (Fig. 2D,D″). In the testis sheath of adult males, Cad-N was distinctly localized in the adjacent membranes of multinucleated myotubes (Fig. 2E).

Hence, we then knocked down *cad-N* by RNA interference (RNAi) with the driver line UAS-Dcr-2;;Mef2-Gal4 specifically in myoblasts. This resulted in a disturbed morphology of the testis. In the wild type, the adult testis is a long, thin tubule of 2.5 coils (Fig. 2F). In the RNAi-mediated knock-down of *cad-N*, the adult testis was partly irregular in shape and had roughly only one coil (Fig. 2G). The testis muscle sheath exhibited holes, which indicated that the myotubes were not properly attached to one another (Fig. 2G′). Genital discs 24 h APF exhibit Duf-expressing FC-like and sticks and stones (SNS)-expressing FCM-like myoblasts on prospective seminal vesicles (Kuckwa et al., 2016). In differential interference contrast (DIC) images, FC-like and FCM-like cells are visible (Fig. S2A), Cad-N expression is largely restricted to FC-like myoblasts (Fig. S2A′). *mefGAL4*-driven knock-down of Cad-N was efficient in FC-like myoblasts, whereas expression was hardly affected in myoblasts lying over the paragonia (compare Fig. S2B′ to wild type Fig. S2A′). At 44 h APF, *cad-N* knock-down testes displayed nascent myotubes, which were distributed all over the testes (Fig. S2C′). In contrast to wild-type testes (Fig. S2C), the nascent myotubes in these *cad-N* knock-down testes are less elongated and their number seemed to be reduced (Fig. S2C). Nevertheless, adult males with this phenotype were able to produce offspring (89%, Fig. S1).

Furthermore, we down-regulated Armadillo (Arm), the beta-catenin homologue in *Drosophila* (Peifer et al., 1992), specifically in myoblasts. Adult testes with down-regulated *arm* had about two coils (Fig. 2H). The testis muscle sheath exhibited numerous small holes, which indicated that the adhesion between single myotubes was disturbed (Fig. 2H′). These males also had a reduced fertility (60%, Fig. S1). Cad-N expression between adjacent myotubes was preserved upon *arm* knock-down, whereas no Cad-N expression was detected when testis myotubes did not adhere to each other (Fig. 2I).

We conclude that Cad-N in cooperation with Arm is involved in testis myotube migration and is necessary both to stabilize the testis muscle sheath and to shape the testis.

### Non-muscle myosin II regulates the migration of nascent myotubes onto the testes

Many migratory processes depend on the actin-myosin network (Campellone and Welch, 2010; Vicente-Manzanares et al., 2009). Using myoblast-specific RNAi experiments, we therefore investigated the relevant myosins of nascent myotubes as they populated the testis. Down-regulation of the light or heavy chain of non-muscle myosin II led to drastic defects.

**Fig. 3.**
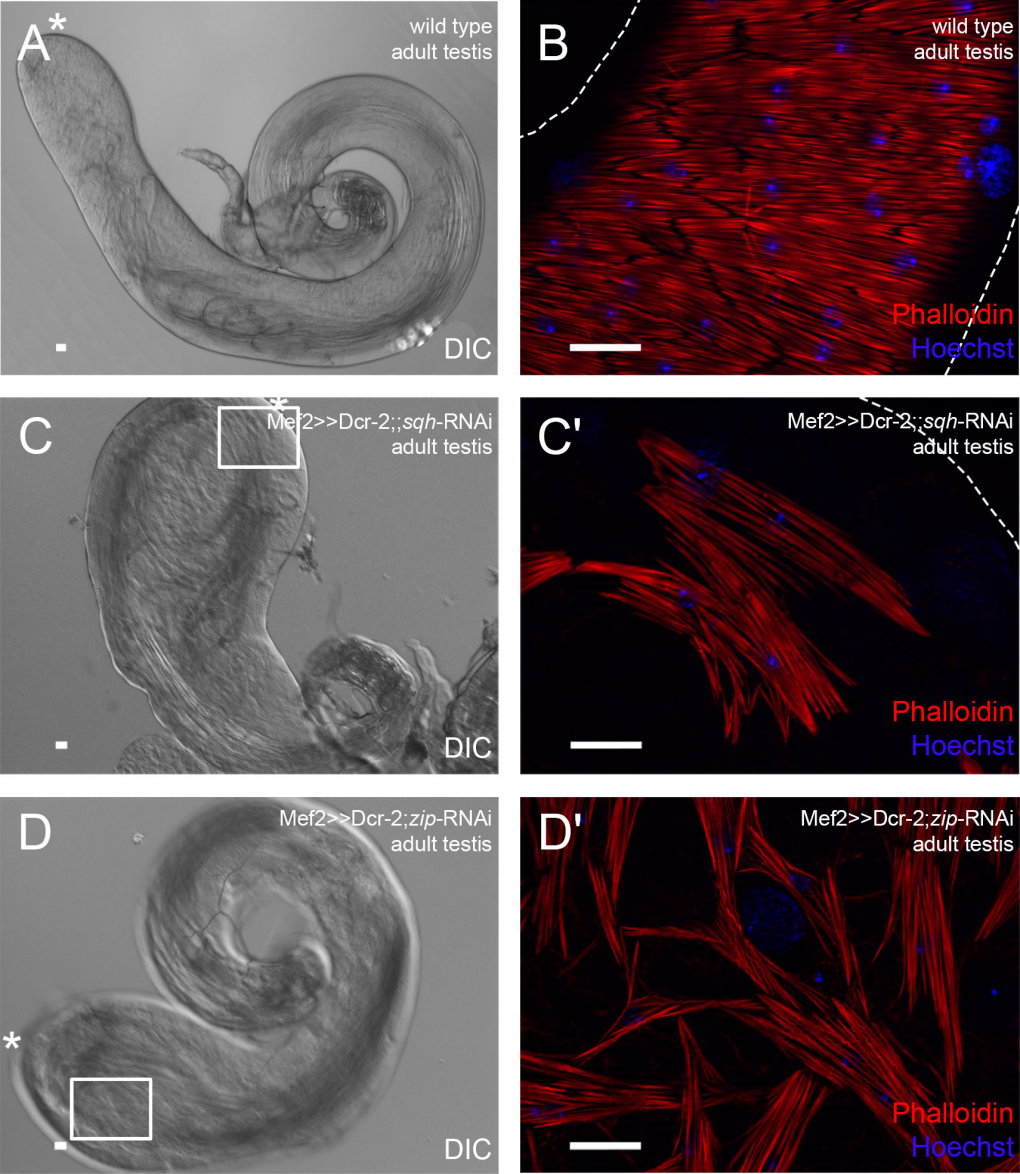
**Myoblast-specific down-regulation of non-muscle myosin II leads to inefficient population of the testis with muscles.** Analysis of adult (A,B) wild-type testes, (C,C′) *sqh* knock-down testes*,* and (D,D′) *zip* knock-down testes. (A,C,D) DIC micrograph; asterisk, hub region. (B,C′,D′) Phalloidin staining to visualize F-actin (red), and Hoechst staining of nuclei (blue). C′ and D′ are enlargements of areas boxed in C and D, respectively. Dotted lines reflect approximate shape of the organ. Scale bars: 20 µm.

When we myoblast-specifically knocked down *spaghetti squash* (*sqh*), the regulatory light chain of non-muscle myosin II (Karess et al., 1991). The testes were smaller than in the wild type (Fig. 3A), and had bulky tips and 1.5 coils in adult males (Fig. 3C). The muscle sheath did not cover the entire testes and had numerous holes; single myofibers appeared shorter (Fig. 3C′) than in the wild type (Fig. 3B). These males had reduced fertility (43%, Fig. S1).

Testes morphology and musculature were also disturbed when *zipper* (*zip*), the non-muscle myosin II heavy chain (Mansfield et al., 1996), was down-regulated. Adult testes had 1.5 coils and a bulky head (Fig. 3D), comparable to the phenotype observed in the *sqh* knock-down, and the muscles resembled a broad-meshed net rather than a sheath (Fig. 3D′). In contrast to *sqh* knock-down males, the fertility of *zip* knock-down males was preserved (100%, Fig. S1). This was surprising because the *sqh* and *zip* knock-down phenotypes resembled each other and are components of myosin II. The observed differences in male fertility might be due to an unpredicted off-target in the sqh RNAi line.

As an example, we first analyzed adult *sqh* knock-down testes in more detail. Cad-N was expressed between the few remaining adjacent myotubes (Fig. S2D), which suggested that the muscle sheath defect was not due to faults in Cad-N-mediated attachment of single myotubes. Instead, it was likely caused by insufficient population of the testis with muscles. We further analyzed the status of the extracellular matrix by monitoring Terribly reduced optic lobes (Trol) (Voigt et al., 2002), which is expressed in the testis muscle sheath (Susic-Jung et al., 2012). Expression of Trol revealed no changes in adult *sqh* knock-down testes (Fig. S2E,F), which indicated extracellular matrix integrity. At 44 h APF, pupal testes with myoblast-specific *sqh* knock-down were populated with fewer nascent myotubes than wild-type testes (Fig. S2H, compare to G). However, the testis shape was not affected at this time of development.

Taken together, these data led us to conclude that non-muscle myosin II is required for testis myotube migration and that correct shaping of the testis depends on the presence of an intact tight muscle sheath.

### DWnt2 signaling controls determination of testis-relevant myoblasts

**Fig. 4.**
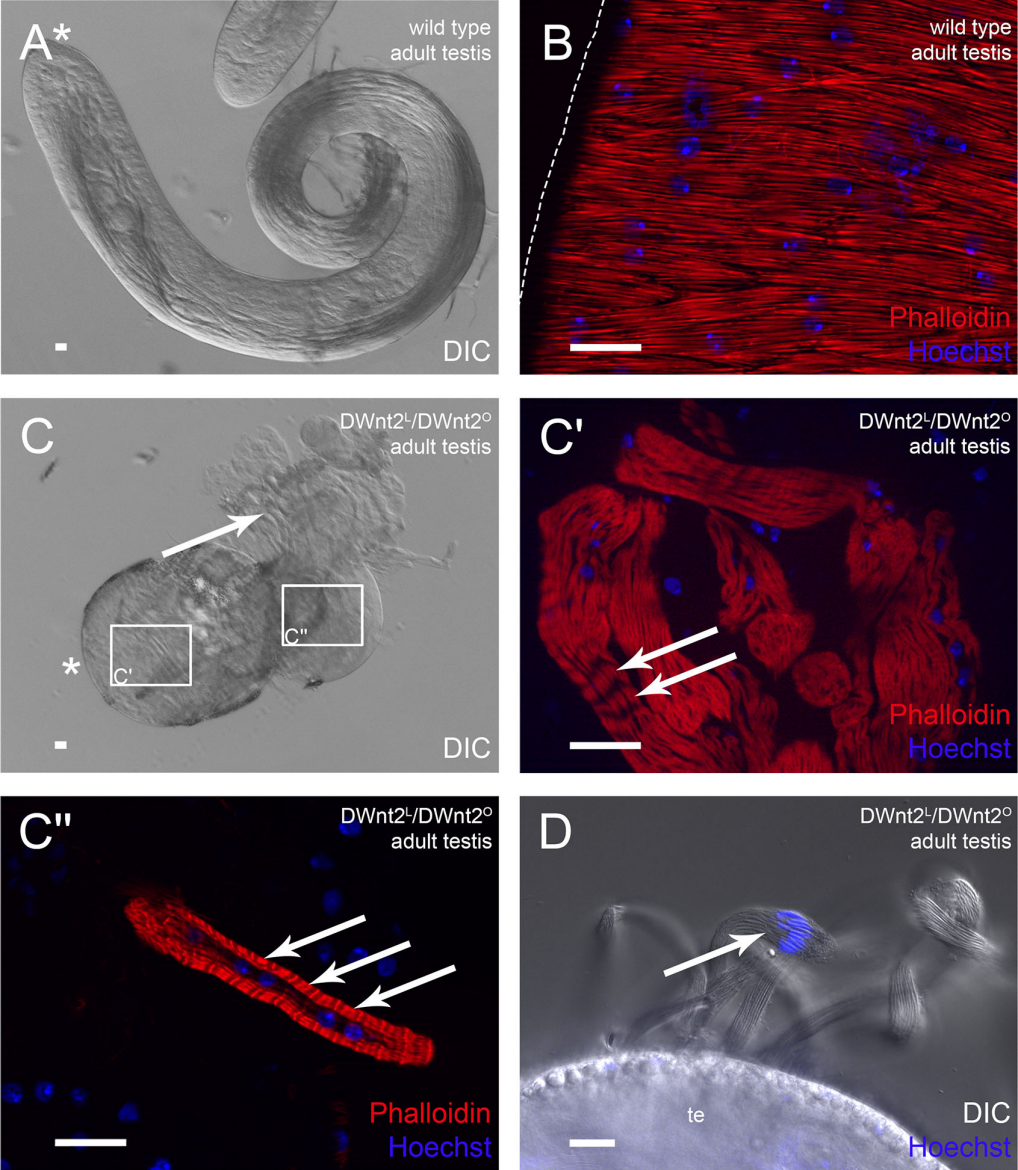
**DWnt2 affects testis muscle determination.** Analysis of adult (A,B) wild-type testes and (C-D) DWnt2^L^/DWnt2^O^ testes. (A,C) DIC micrographs; arrow, leaking sperm; asterisk, hub region. (C′,C″) Enlargement of the respective boxed areas in C stained with Phalloidin to visualize F-actin (red) and Hoechst to visualize nuclei (blue). Arrows, thin actin filaments. (D) DIC micrograph and Hoechst staining (blue) of adult DWnt2^L^/DWnt2^O^ testis. Arrow, nuclei of leaking spermatid bundles. Scale bars: 20 µm.

It has been previously suggested that DWnt2 plays a role in the migration of myoblasts from the prospective seminal vesicle onto the testes (Kozopas et al., 1998). Therefore, we analyzed the testis shape and muscle sheath of two amorphic *DWnt2* alleles in *trans* (*DWnt2^L^*, *DWnt2^O^*; Kozopas et al., 1998). The resulting flies were not able to hatch, and pharate lethal. The development and morphology of testes was variable in these flies, as has also been recently observed by Linnemannstöns et al. (2014). The most abundant phenotypes were deformed and not elongated adult testes that did not resemble the wild-type adult testis shape (Fig. 4A). Instead, the shapes were comparable to those of pupal testes (Fig. 4C). Testis stability was decreased, and germ cells leaked (Fig. 4C, arrow). Unlike the wild-type musculature (Fig. 4B), smooth-like muscles were not detected on these testes, but some multinucleated actin-rich structures that contained repetitive actin filaments resembling striated muscles were visible (Fig. 4C′,C″, arrows). The leaking cysts with elongated spermatids that appeared shortly before individualization (Fig. 4D, arrow) indicated that germ cell maturation proceeded normally. This is in agreement with our earlier observations that spermatogenesis *in vitro* in cultured isolated cysts proceeds until shortly before individualization, independently of the presence of the testis muscle sheath (Awe and Renkawitz-Pohl, 2010; Gärtner et al., 2014). Other organs of the reproductive system showed no obvious defects (data not shown), which suggested a selective effect on testis-relevant myoblasts.

From these data, we concluded that *DWnt2^L^*/*DWnt2^O^* mutant flies fail to determine testis-specific myoblasts from the common pool of myoblasts. However, this does not exclude an additional later role of DWnt2 during migration of muscles onto the testes.

### Heartless-dependent FGF signaling is essential for the migration of nascent myotubes onto the testis

**Fig. 5.**
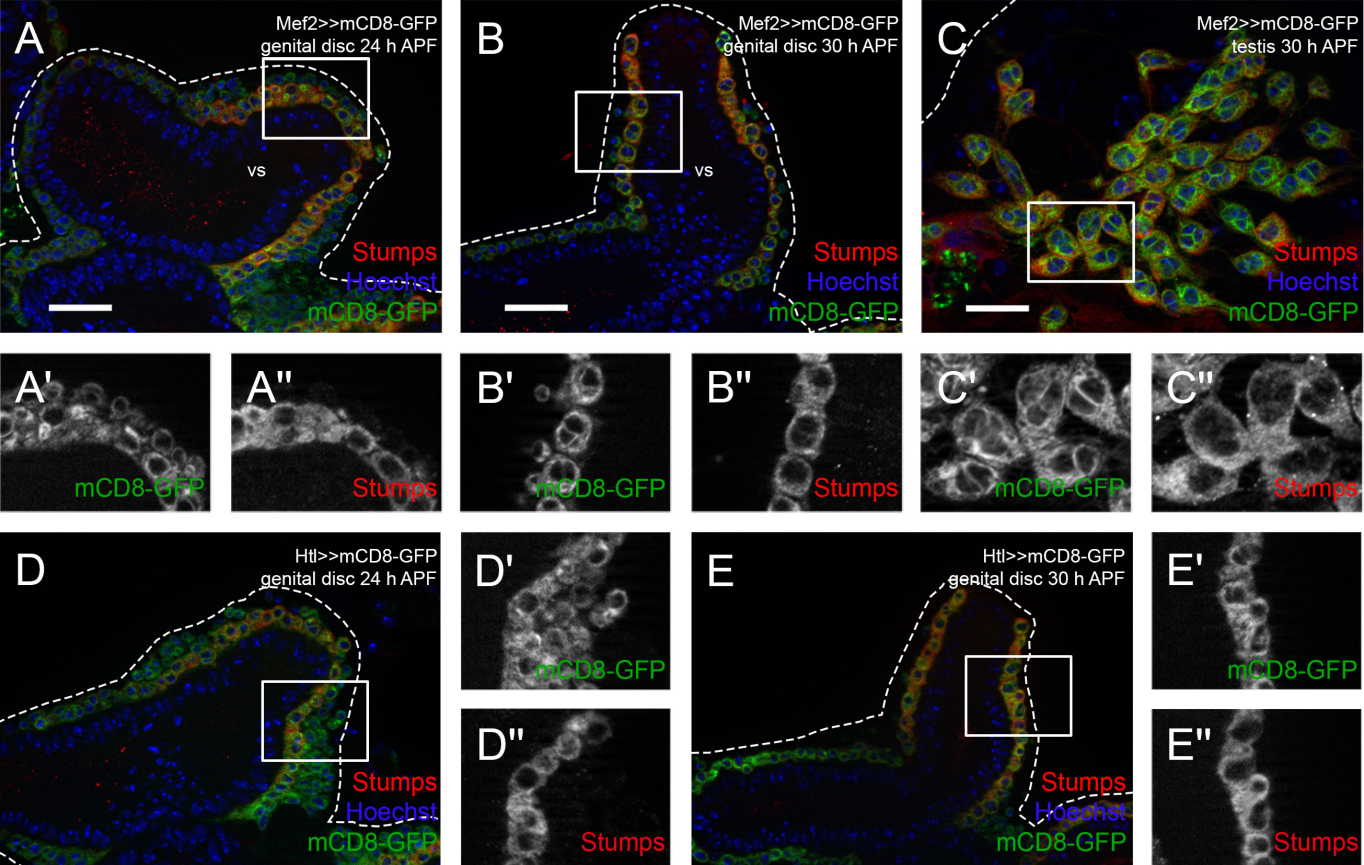
**FGF signaling components are expressed during migration of testis myotubes.** Immunofluorescence analysis of Stumps. Myoblasts and nascent myotubes on (A-A″) wild-type genital discs 24 h APF, (B-B″) wild-type genital discs 30 h APF (magnifications of prospective seminal vesicles are shown), and (C-C″) pupal testis 30 h APF stained or marked with anti-Stumps (red), Mef2-driven mCD8-GFP (green), and Hoechst (blue; nuclei). (A′,A″,B′,B″,C′,C″) Enlargement of boxed areas in A, B, and C, respectively, marked with GFP or stained with anti-Stumps. (D-E″) Myoblasts and myotubes on Htl-Gal4≫mCD8-GFP genital discs at (D-D″) 24 h APF (magnification of prospective seminal vesicle is shown) and (E-E″) 30 h APF, stained or marked with anti-Stumps (red), Htl-driven mCD8-GFP (green), and Hoechst (blue). (D′,D″,E′,E″) Enlargement of boxed area in D and E, respectively, marked with Htl-driven mCD8-GFP or stained with anti-Stumps. Dotted lines reflect approximate shape of the organ. vs, seminal vesicle. Scale bars: 20 µm.

FGF signaling is involved in various migration processes throughout the animal kingdom (Itoh and Ornitz, 2011; Ornitz and Itoh, 2015; Shilo, 2016). Therefore, we examined the role of FGF signaling in the migration of nascent testis myotubes. We analyzed the expression of Stumps, the intracellular adaptor protein specific for FGFRs, which is also known as Heartbroken (Hbr) and Downstream of FGF (Dof) (Imam et al., 1999; Michelson et al., 1998a; Vincent et al., 1998), using a specific antibody (Vincent et al., 1998) on genital discs. At 24 h APF, Stumps was detected in the cytoplasm of myoblasts in the inner layer, the so-called FC-like myoblasts (Fig. 5A–A″). Nascent myotubes on genital discs 30 h APF (Fig. 5B–B″) and testes 30 h APF (Fig. 5C–C″) also expressed Stumps in the cytoplasm. We investigated the potential expression pattern of Htl on male genital discs in an Htl-Gal4 driver line that has been shown to drive expression in the epithelial sheath of the ovary (Irizarry and Stathopoulos, 2015). We observed Htl-driven mCD8-GFP expression in myoblasts on the prospective seminal vesicles at 24 h APF (Fig. 5D) and 30 h APF (Fig. 5E). Notably, Stumps was expressed in FC-like Htl-positive myoblasts on developing seminal vesicles at 24 h APF (Fig. 5D–D″) and in all nascent myotubes at 30 h APF (Fig. 5E–E″), which suggested that FGF signaling might be activated during migration.

**Fig. 6.**
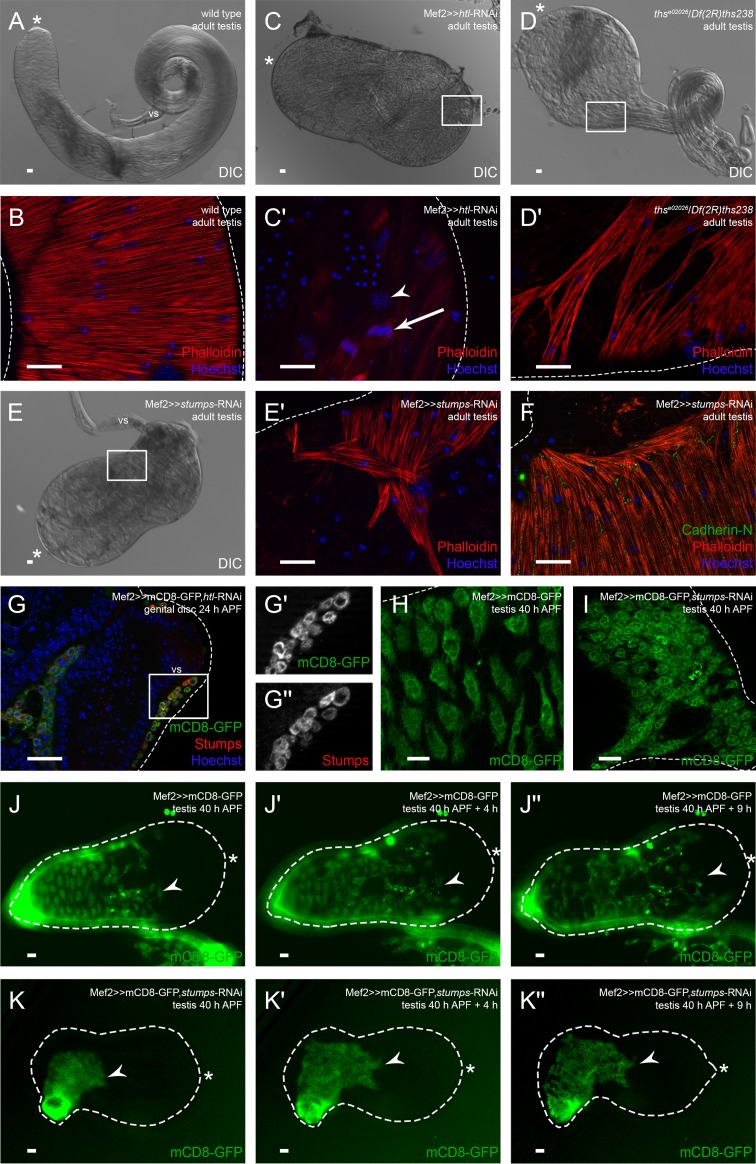
**Ths-activated Heartless is essential for populating the testis with myotubes.** Analysis of *htl* and *stumps* knock-down and *ths* mutant. (A) DIC micrograph of adult wild-type testis. (B) Phalloidin staining to visualize F-actin (red), and Hoechst staining of nuclei (blue). (C) DIC micrograph of adult *htl* knock-down (v6692) testis. (C′) Enlargement of boxed area in C showing adult *htl* knock-down testis stained with Phalloidin (red; F-actin) and Hoechst (blue; nuclei); arrowhead, pigment cell nuclei; arrow, spermatids during individualization. (D) DIC micrograph of adult *ths* mutant testis. (D′) Enlargement of boxed area in D showing adult *ths* mutant testis stained with Phalloidin (red) and Hoechst (blue). (E) DIC micrograph of adult *stumps* knock-down testis. (E′) Enlargement of boxed area in E showing adult *stumps* knock-down testis stained with Phalloidin (red) and Hoechst (blue). (F) Adult *stumps* knock-down testis stained with anti-Cad-N (green), Phalloidin (red), and Hoechst (blue). (G-G″) *htl* knock-down genital disc 24 h APF stained or marked with anti-Stumps (red), GFP (green), and Hoechst (blue); magnification of prospective seminal vesicle is shown. (G′,G″) Enlargement of boxed area in G marked with GFP or stained with anti-Stumps. (H) Myoblasts on wild-type testis 40 h APF marked with Mef2-driven mCD8-GFP (green). (I) Myotubes of *stumps* knock-down testis 40 h APF marked with GFP (green). (J-K″) Live imaging over time of testes 40 h APF expressing Mef2-driven mCD8-GFP to reveal the migration of nascent myotubes in an *ex vivo* culture of (J-J″) wild-type testis and (K-K″) *stumps* knock-down testis. Dotted lines reflect the approximate shape of the organ. Arrowheads, the front of migrating nascent myotubes; asterisk, hub region; vs, seminal vesicle. Scale bars: 20 µm.

In *Drosophila*, Stumps is a unique adaptor protein in FGF signaling that can be activated by two FGFRs (Vincent et al., 1998). Myoblast-specific knock-down of the FGFR Btl did not result in any defects in testis musculature or shape, but resembled the wild-type situation (Fig. S3A). By contrast, we observed very drastic defects when the FGFR Htl was knocked down. Specifically, all flies were pharate lethal, and most testes were not attached to the seminal vesicles and did not elongate (Fig. 6C). The testes did not contain muscles, and the sheath consisted solely of pigment cells (Fig. 6C′, arrowhead). Nevertheless, spermatogenesis appeared to be normal in these testes since we observed bundles of spermatids shortly before or during individualization (Fig. 6C′, arrow). This indicates that the presence of testis muscles is not essential for germ cell maturation, as we also observed in hypomorph DWnt2 alleles (Fig. 4). In the *htl* knock-down, the other organs of the male reproductive system were malformed and partly degraded, and muscles were mainly absent (data not shown). This might be due to myoblast determination defects, since *htl* knock-down genital discs 24 h APF contained fewer myoblasts than wild-type genital discs 24 h APF. This was especially the case in the posterior part of the genital discs, where myoblasts for the muscle sheaths of the ejaculatory duct and sperm pump are localized (Fig. S3B,C, arrows). At 30 h APF, prospective seminal vesicles of *htl* knock-down genital discs contained fewer nascent myotubes than the wild type (compare Fig. S3F-F″ to Fig. 5B). This is in agreement with the observed Htl-driven expression of mCD8-GFP in most myoblasts on genital discs 24 h APF (Fig. S3D). Myoblast-specific expression of a dominant-negative (DN) version of Htl lacking the kinase domain (Michelson et al., 1998b) resulted in a milder phenotype than the *htl* knock-down, comparable to the other *htl*-RNAi line (Table S1). The adult testes were elongated and coiled, but had bulky tips as observed with the DN-version of Htl (Fig. S3E). The testis muscles covered the organs except for the tips (Fig. S3E′). To gain further insight into which FGF ligand activates Htl in nascent myotubes, we analyzed the testis shape and musculature of hypomorph *ths^e02026^*/*Df(2R)ths238* mutants. Adult testes had approximately two coils and a very bulky tip (Fig. 6D). The smooth-like testis muscle sheath had numerous holes, and the testis tip was free of muscles (Fig. 6D′) as previously observed for DN-version of Htl and knock down by the RNAi line BL35024. Unfortunately, we could not analyze the other FGF ligand Pyr because no hypomorph alleles were available.

When we down-regulated *stumps* specifically in myoblasts, a strong phenotype was generated, comparable to that of the *htl* knock-down. But unlike the strongest *htl* knock-down testes, adult testes with reduced Stumps levels were attached to seminal vesicles, and about 30% of the testis sheath contained muscles. All flies were pharate lethal, and most testes attached to the seminal vesicles did not elongate (Fig. 6E). The muscles did not build a complete sheath but remained at the basal end of the testis, and the muscle pattern was disturbed (Fig. 6E′). On the adult *stumps* knock-down testis, Cad-N was normally expressed in the membranes between adjacent myofibers, where muscles formed an intact sheath (Fig. 6F). However, where myofibers did not attach to one another, no Cad-N was detected (Fig. 6F). No Stumps expression was visible in *stumps* knock-down genital discs 24 h APF, which indicated a strong down-regulation of *stumps* (Fig. S3G-G″). After *stumps* knock-down – in contrast to the *htl* knock-down (Fig. S3F-F″) – we observed many myoblasts lying over the seminal vesicles (Fig. S3G-G″). Stumps expression on *htl* knock-down genital discs 24 h APF remained unchanged in testis-relevant myoblasts (Fig. 6G-G″). At 40 h APF, mCD8-GFP-positive nascent myotubes were visible on both wild-type (Fig. 6H) and *stumps* knock-down testes (Fig. 6I), which indicated that the initial migration process was intact upon *stumps* knock-down.

These results suggest that Htl-controlled FGF signaling activated by Ths is essential for the correct migration of nascent myotubes on testes.

### Stumps is necessary for proper myotube migration on the testis *ex vivo*

To gain further insights into the migration process on the testis, we established *ex vivo* live imaging of co-cultures of pupal testes and genital discs. For investigating the migrating nascent myotubes on the testis itself, we used 40 h APF testes expressing myoblast-specific mCD8-GFP. The testes were still attached to part of the developing seminal vesicles, but not to the genital disc. When live imaging started, approximately 60% of the pupal testis surface was already covered with multinucleated nascent myotubes (Fig. 6J, arrowhead). Four hours later, the myotubes had spread out and migrated towards the apical region (Fig. 6J′, arrowhead). After nine hours, myotubes covered about 80% of the pupal testis surface (Fig. 6J″, arrowhead). During live imaging, the testis grew in length.

We then tested the effect of down-regulated *stumps* on the migration process on pupal testes. We cultured *stumps* knock-down testes 40 h APF expressing myoblast-specific mCD8-GFP. These testes were also still attached to a part of the developing seminal vesicle. When live imaging started, approximately 30% of the pupal testis was covered with nascent myotubes (Fig. 6K, arrowhead). After four hours, myotubes covered about 40% of the testis (Fig. 6K′, arrowhead). After nine hours, myotubes still covered no more than 40% of the testis (Fig. 6K″, arrowhead). Strikingly, the morphology of the nascent myotubes was affected; the cells seemed smaller than in the control (Fig. 6K, compare to J) and did not spread out on the testis during live imaging. In addition, the testis grew only little in length.

From these results, we conclude that Stumps is essential for the migration of nascent myotubes on pupal testes, and that when Stumps is efficiently reduced, testes can grow and even attach to the developing seminal vesicles, but nascent myotubes fail to fully cover the testes.

### Non-muscle myosin II expression depends on Heartless signaling

So far, we gained evidence for Heartless-dependent migration of nascent myotubes along the testes and showed that knock-down of Cad-N (Fig. 2G-G′) and Sqh (Fig. 3) lead to distortion of the coverage of testes with muscles. We now asked whether the expression of Cad-N or Sqh or both depends on Heartless signaling. As the *htl*-RNAi line with the strongest phenotype after myoblast-specific activation had no muscles on the testis, we applied a second *htl*-RNAi line (BL35024) in our analysis, which resembles the Htl-DN (Fig. S3E) phenotype and the *thisbe* hypomorph mutant and thus had muscles on the testes. As RNAi-mediated knock-down of Stumps (Fig. S3G-G′) was very efficient (Fig. S3G-G″), we focused on *stumps* in our analysis in parallel to the *htl-*RNAi line BL35024.

**Fig. 7.**
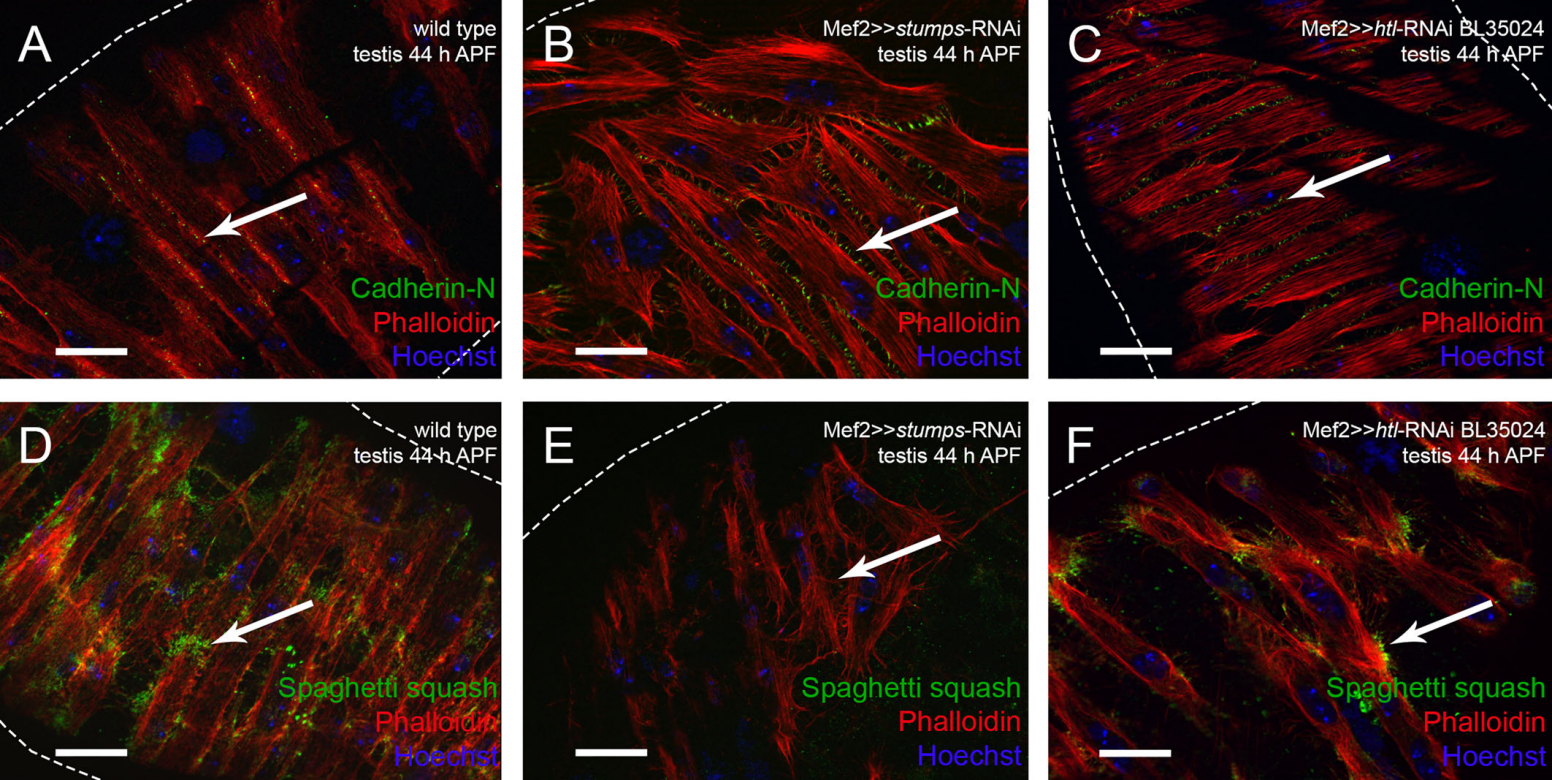
**Expression of the myosin II subunit Spaghetti squash is not detectable after knock-down of Stumps.** (A-F) Visualization of muscles on testes by Phalloidin (red). (A-C) Expression of Cad-N in (A) wild type, (B) *stumps* knock-down, and (C) *htl* knock-down. (D-F) Sqh expression in (D) wild type (Z-stacks, 27 images with 0.48 µm per layer), (E) *stumps* knock-down (Z-stacks, 12 images with 0.52 µm per layer), and (F) *htl* knock-down (Z-stacks, 9 images with 0.24 µm per layer). Arrows in A-C indicate Cad-N and in D-F indicate Sqh; dotted lines indicate border of testis.

We observed Cad-N expression in nascent myotubes migrating along the testis and in the mature musculature (Fig. 2D-E). We asked whether reducing Heartless signaling impairs expression of Cad-N in pupal testes (arrows in Fig. 7A-C). Cad-N was expressed at the site of contact (Fig. 7A) in wild type and after knock-down of either Htl (Fig. 7C) or Stumps (Fig. 7B), which indicates that Cad-N expression is independent of Htl signaling. At 44 h APF in the Stumps knock-down mutant, nascent myotubes were less tightly packed and many Cad-N-positive filopodia were visible at the contact sites between neighboring myotubes (Fig. 7B).

Next we asked whether Sqh, the light chain of myosin II, is expressed dependent on Htl signaling. At 44 h APF, Sqh localized to the filopodia of nascent myotubes on wild-type testes, and Sqh was enriched at the ends of stretching myotubes (Fig. 7D). In the *htl*-RNAi knock-down situation, however, nascent myotubes were less densely packed and had started to stretch while encircling the testis tube. In this situation, Sqh expression was not obviously disturbed (Fig. 7F). This is not surprising since in the less efficient *htl*-RNAi line showed a weak phenotype comparable to the dominant-negative version. By contrast, we did not detect Sqh in the poorly stretched nascent myotubes after efficient reduction of Stumps in the parallel experiment (Fig. 7E) (for knock down phenotype with respect to testis shape see Fig. 6E). We propose that Heartless signaling via Stumps directly or indirectly activates the synthesis of Sqh, the regulatory light chain of myosin II.

## DISCUSSION

### Wnt signaling does not directly affect testis myotube migration but affects determination of testis-relevant myoblasts

DWnt2 functions in pigment cell determination and possibly also in the migration of nascent myotubes onto pupal testes in addition to, or as a consequence of, the failure of pigment cell migration (Kozopas et al., 1998). We checked whether amorphic DWnt2 alleles have migration defects. In agreement with the results of Kozopas et al. (1998) and Linnemannstöns et al. (2014), an amorphic allelic combination of *DWnt2* produced small testes of various sizes. Kozopas et al. (1998) observed only a small amount of muscles on adult DWnt2 mutant testes. We showed that smooth-like muscles were absent from the testis sheath. Surprisingly, a few striated muscles were visible on these testes.

The results presented here led us to tentatively conclude that DWnt2 affects cell fate determination of testis-relevant myoblasts. Indeed, it has been shown that Wnt signaling specifies cell identity of a subset of somatic muscle founder cells in *Drosophila* embryogenesis (Baylies et al., 1995) and during many other determinations of the fate of the cell (Bertrand, 2016; Munoz-Descalzo et al., 2015). However, an additional later role in the migration of testis myotubes cannot be excluded.

### Multiple roles of Heartless-dependent FGF signaling during the development of the male reproductive system

The FGF receptor Btl is required for migration of glia and tracheal cells (Klämbt et al., 1992). In our study, down-regulation of the FGFR Btl, specifically in myoblasts, did not result in migration defects of testis myotubes. Conversely, knock-down of *htl* led to adult testes of a severely reduced size that remained free of muscles or to less drastic phenotypes, depending on the RNAi fly line chosen.

**Fig. 8.**
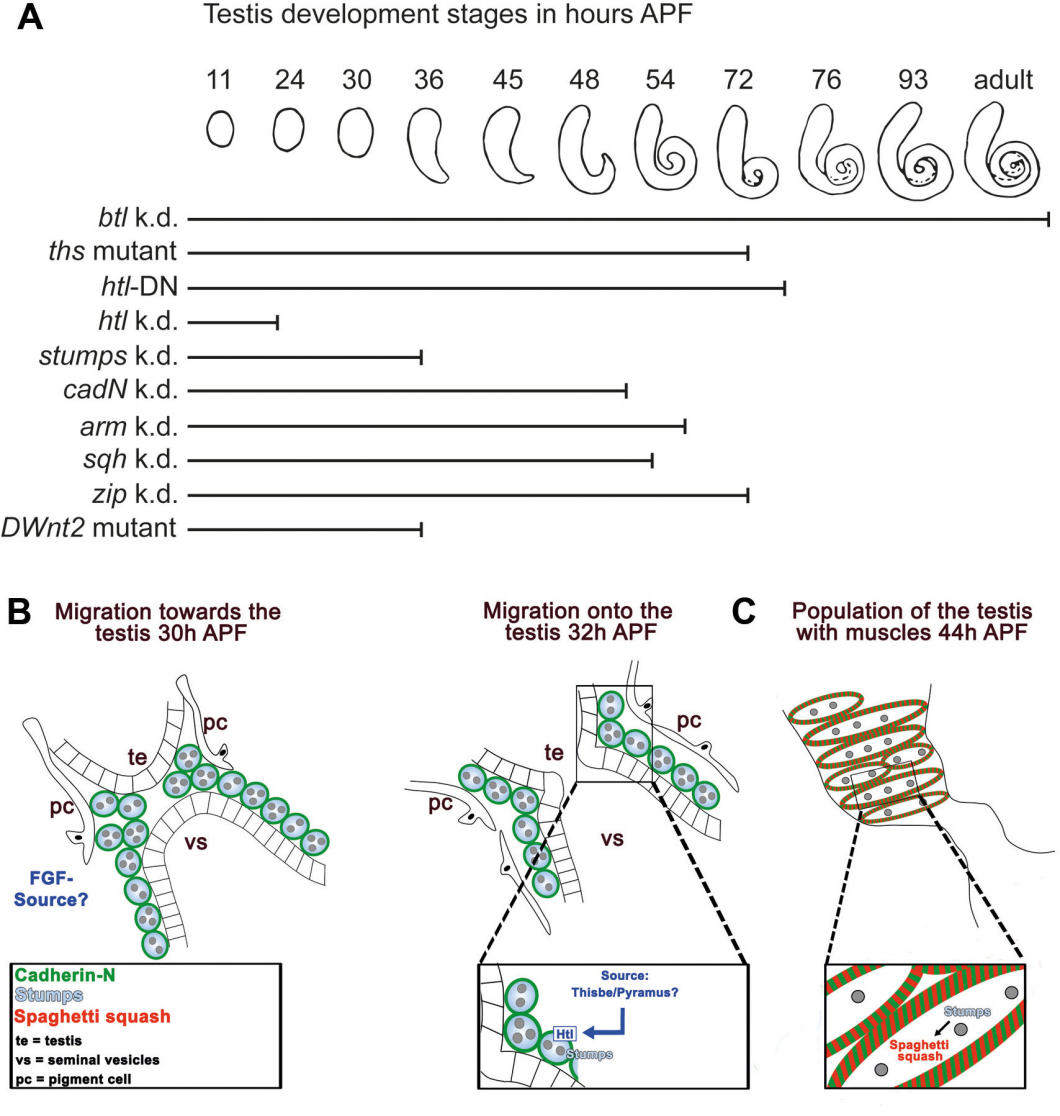
**Model of testis myotube migration.** (A) Summary of shaping defects of knock-down/mutant testes. Stages of testis development in wild-type males during metamorphosis, from 11 h APF to adult. Bars indicate the stage to which to mutant testes develop [myoblast-specific knock-down (k.d.) mutants, mutant testes expressing the dominant-negative (DN) Htl protein, or a DWnt2 mutant]. According to the affected gene or genetic manipulation, testis development correlates to different stages of wild-type development. For example, down-regulation of *btl* does not interrupt testis development, whereas *htl* knock-down leads to a very early stop in development. (B,C) Two-phase model of testis myotube migration. (B) Phase one represents the Htl dependent migration of nascent myotubes onto the testis before and after fusion of the epithelium of the seminal vesicle (vs) and the terminal epithelium (te) of the testes. Pigment cells (pc) migrated towords the vs. The FGF ligand Ths and possibly Pyr are secreted by an unknown source. (C) In phase two, nascent myotubes are already on the testis and migrate further towards the tip. Cad-N mediates adhesion between adjacent myotubes. Stumps might control the migration process, likely via non-muscle myosin II. Sqh expression depends on Stumps, which suggests Sqh regulation by Htl signaling. Note that after an initial phase one, both phases run in parallel until all myotubes reach the testis.

The *htl* knock-down yielded two further phenotypes. One phenotype was the drastically reduced number of non-testes-relevant myoblasts building the genital discs 24 h APF, while testis-relevant myoblasts were present on the developing seminal vesicles. This might be due to decreased proliferation of myoblasts on genital discs, since myoblasts undergo mitosis around 16 h APF (Kuckwa et al., 2016). Alternatively, this might be due to survival or determination defects. The second phenotype was the lack of a connection between the seminal vesicles and the testes. Knock-down of *htl* by one RNAi line caused testes development to stop early, whereas the dominant-negative version of Htl (Fig. 8A) and a weaker RNAi line (BL 35024) resulted in testes shapes that pointed towards a much later stop in development. Testes of hypomorph mutants of the Htl ligand Ths (Fig. 8A) showed a remarkably similar phenotype. This suggests that Ths is an important ligand of Htl in this system.

Stumps was expressed in FC-like myoblasts and nascent myotubes adjacent to the epithelium on genital discs and testes. In addition, down-regulation of the adaptor protein *stumps* yielded drastic defects in covering the testes with myotubes. This could be caused by a reduced number of myotubes or by a migration defect. We observed a fairly normal amount of myoblasts lying over the seminal vesicles. This and the disruption of migration of these myotubes in *ex vivo* cultures of testes argue for a migration defect in addition to morphology defects. *stumps* knock-down pupal testes 40 h APF exhibited many nascent myotubes, which indicated that initial migration occurred. However, we cannot exclude that we were unable to detect a low level of Stumps in immunofluorescence assays and that such a low amount could be sufficient for initial migration onto the testes.

Our results led us to conclude that migration of nascent myotubes on the testis depends on FGF signaling via Ths, Htl, and Stumps. Future research will focus on unravelling the distinct functions of FGF signaling via Heartless during muscle development in the male reproductive system.

### Non-muscle myosin II drives the migration of nascent testis myotubes dependent on Heartless signaling

Down-regulation of non-muscle myosin II (i.e. *zip* or *sqh*) resulted in adult testes that were not fully covered with muscles, which indicated that non-muscle myosin II is necessary for the migration of nascent testis myotubes. Myosin II is also required for proper detachment and migration of border cells during egg chamber development (Combedazou et al., 2016; Majumder et al., 2012), and *Drosophila* tubulogenesis (Saxena et al., 2014; Nie et al., 2014). Signaling through epidermal growth factor receptor activates non-muscle myosin II (Saxena et al., 2014; Combedazou et al., 2016). Here, we propose that Heartless-mediated FGF signaling directly or indirectly induces non-muscle myosin II, which promotes migration of nascent testis myotubes. This might explain migration and morphological defects of myotubes *in vivo* and *ex vivo*.

### Cadherin-N and Armadillo are expressed during cell migration and for stabilizing the testis muscle sheath

Cad-N was expressed in myoblasts and nascent myotubes of genital discs and pupal testes as well as in the membranes of adult testis myotubes. In contrast to the epithelial-mesenchymal transition during embryogenesis (Leptin and Grunewald, 1990), no change in expression from Shg to Cad-N was observed when the nascent myotubes started to migrate. During the initial migration onto and along the testis, Cad-N was expressed, which suggests a collective mode of cell migration. We propose that lack of Cad-N or Arm leads to disturbances in collective cell migration. As a consequence, far fewer myotubes spread over the testes, and residual myotubes cannot yield a tight sheath of muscles. This leads to thickened areas in the testes, where the population of muscles is low. Therefore, we in turn propose that Cad-N in cooperation with Arm is furthermore essential for mediating cell adhesion between single testis myotubes, thereby stabilizing the testis muscle sheath and the tubular and coiled shape of the testes. In contrast, to Sqh we gained no evidence for Htl dependent expression of Cad-N.

### Testis shaping depends on an intact muscle sheath

Shaping of adult testes was disturbed to varying degrees depending on the genetic background (Fig. 8). The shape resembled that of different developmental time points in the wild type (Fig. 8A). Mutants with amorphic DWnt2 alleles, *htl* knock-down, or *stumps* knock-down showed hardly any shaping. These phenotypes correlate with the total absence or presence of only a few testis muscles. Hypomorph Ths mutants as well as the expression of a dominant-negative version of Htl led to a less severe phenotype that matched the shaping defects produced by down-regulating *htl* with another RNAi fly line. These adult testes resembled those of the wild type at 72 h APF. Knock-down of *cadN*, *arm*, *sqh*, or *zip* led to adult testes whose shape, but not the degree of coverage of the testes with muscles, resembled that of wild-type testes at 54–72 h APF. At 42 h APF, nascent myotubes reach the testis tip in the wild type (Kuckwa et al., 2016). This is also the case when *cadN*, *arm*, *sqh*, or *zip* is down-regulated specifically in myoblasts. However, already at this stage, fewer myotubes were present on knock-down testes (Fig. 7), which supports our conclusions that Cad-N and non-muscle myosin II are required for the migration of testis myotubes and that an intact muscle sheath is required for the shaping of the testis.

### Conclusions and model

Based on our results, we propose a model in which testis myotube migration is divided into two Htl- and Cad-N-dependent phases. (i) After myoblast fusion, when Stumps and Cad-N are distributed along the plasma membrane of nascent testis myotubes that are in close contact to the adjacent epithelium of the prospective seminal vesicles, FGF signaling initiates collective migration of nascent testis myotubes from the prospective seminal vesicle onto the testis (Fig. 8B). (ii) The coverage of the testes with muscles is achieved by collective cells migrating towards the testis tip, and this migration requires myosin II and the formation of a network of nascent myotubes with Cad-N-positive connections (Fig. 8C).

We propose that the ligand Ths and possibly also Pyr binds Htl in nascent myotubes and thereby activates FGF signaling via Stumps during both phases of migration; the source of these ligands needs to be determined. Downstream, components of MAPK signaling, such as Erk, could be activated, which might cause changes in transcription of different target genes. Indeed, we observed Htl-dependent expression of Sqh. We hypothesize that Thisbe/Heartless-induced MAPK signaling might link to the actin cytoskeleton via non-muscle myosin II, thereby facilitating cytoskeletal changes needed for cell migration (Fig. 8B).

Our data provide first insights into the Thisbe/Heartless-dependent regulation of myotube migration via myosin II expression from the seminal vesicle to and along the testes, which is dependent on successful connection of these tissues. Future studies aiming at identifying missing components and functions to fully elucidate testis myoblast specification and myotube migration will require the establishment of more specific tools, such as driver lines specifically active in testes myoblasts and myotubes.

## MATERIALS AND METHODS

### Fly stocks

Flies were kept and RNAi crossings were carried out on standard medium at 25°C. w^1118^ (BL6326) was used as the wild-type reference. The following transgenic flies were used: Mef2-Gal4 (Ranganayakulu et al., 1995), Htl-Gal4 (BL40669), UAS-Dcr-2;;Mef2-Gal4 (BL25756), UAS-htl-DN (BL5366), and UAS-mCD8-GFP (BL32186). For RNAi experiments, the following fly lines were used: UAS-*cadN*-RNAi (v1092, v1093, v101642), UAS-*arm*-RNAi (BL31304, BL35004), UAS-*sqh*-RNAi (BL32439, BL33892), UAS-*zip*-RNAi (BL36727, BL37480), UAS-*btl*-RNAi (BL40871, v27106), UAS-*htl*-RNAi (BL35024, v6692), and UAS-*stumps*-RNAi (v21317, v105603). We conducted all RNAi experiments with at least two different fly lines per gene; the differences were in knock-down efficiencies or effects of second site insertions, as described in Vissers et al. (2016). An overview of all produced phenotypes is given in Table S1. The *ths* mutant was generated by crossing *ths^e02026^*/CyO.actin-GFP (Stathopoulos et al., 2004) and *Df(2R)ths238*/CyO.actin-GFP (Kadam et al., 2009). For DWnt2 experiments, DWnt2^L^ (BL6909, Kozopas et al., 1998) and DWnt2^O^ (BL6958, Kozopas et al., 1998) flies were used. BL flies were ordered from Bloomington *Drosophila* Stock Center (Bloomington, IN, USA), and v flies were obtained from Vienna *Drosophila* RNAi Center (Vienna, Austria).

### Fertility tests

One adult male of the F_1_ generation from RNAi crossings was mated with three virgin females (w^1118^) over seven days. After two weeks, the efficiency of the matings was examined. All fertility tests were carried out at 25°C.

### Immunofluorescence

Pupae selection, genital disc dissection, and immunofluorescence were carried out as described elsewhere (Kuckwa et al., 2016) and were repeated at least three times. The following antibodies were used: anti Duf/Kirre (1:500, Kreisköther et al., 2006), anti-Cadherin-N (1:100, DSHB DN-Ex #8), anti-Shotgun (1:100, DSHB DCAD2), anti-Stumps (Dof, 1:1000; gift from Maria Leptin, University of Cologne, Germany; Vincent et al., 1998), antiSqh [1:10, 64 h incubation time; anti Phospho-Myosin Light Chain 2 (ser19), #3671 Cell Signaling; dilution according to Saxena et al., 2014; Nie et al., 2014] and anti-Trol (Perlecan domain V, 1:2000; gift from Stefan Baumgartner, Lund University, Sweden; Friedrich et al., 2000). The following secondary antibodies were used: anti-rat Cy3 (1:500; Jackson ImmunoResearch Laboratories), anti-rat Alexa Fluor^®^ 488 (1:500; Jackson ImmunoResearch Laboratories), anti-rabbit DyLight 488 (1:500; Vector Laboratories), anti-rabbit DyLight 549 (1:500; Vector Laboratories). For visualization of F-actin, we used Phalloidin-Atto 565 (4 nmol l^−1^; 94072, Sigma-Aldrich, St. Louis, MO, USA); to visualize nuclei, Hoechst 33342 was used (3.2 µg ml^−1^; 62249, Thermo Fisher Scientific, Waltham, MA, USA).

### Live imaging of *ex vivo* cultures

Male white prepupae were collected at 0 h APF and aged on a moistened filter. Genital discs and pupal testes were dissected in culture medium and transferred into a tissue culture treated µ-Dish^35mm,high^ (81156, ibidi, Martinsried, Germany) containing 2 ml culture medium Shields and Sang M3 Insect Medium (S8398, Sigma-Aldrich) was made according to Aldaz et al. (2010) by filtering and supplementing with 2% fetal bovine serum (S0113, Biochrom AG, Berlin, Germany), 0.5% penicillin-streptomycin (P11-010, PAA, Cambridge, UK), and 0.1 µg ml^−1^ Ecdysone (H5142, Sigma-Aldrich). Culture medium worked successfully for up to 4 days when stored at 4°C. A Zeiss AxioObserver Z.1 inverse microscope was used for live imaging. A Z-stack was acquired every 15 min for up to 10 h.

### Image acquisition and processing

Conventional fluorescent images and optical sections were captured with a Zeiss AxioObserver Z.1 inverse microscope with ApoTome function. Images were taken and processed with AxioVision LE64 (Carl Zeiss Microscopy GmbH, Jena, Germany), figures were assembled with Adobe Photoshop CS6 (Adobe Systems Incorporated, San José, CA, USA), and models were generated in Adobe Illustrator CS6 (Adobe Systems Incorporated). Charts were generated in Microsoft Excel 2016 (Microsoft Corporation, Redmond, WA, USA).

## Supplementary Material

Supplementary information
