# Supplementary material for: Myotube migration to cover and shape the testis of Drosophila depends on Heartless, Cadherin/Catenin, and myosin II
Source: Biol Open. 2017 Nov 9;6(12):1876–88. doi: 10.1242/bio.025940 (PMC5769643; doi:10.1242/bio.025940)
Supplement: Supplementary information [file biolopen-6-025940-s1.pdf]

Suppl Figures and Tables

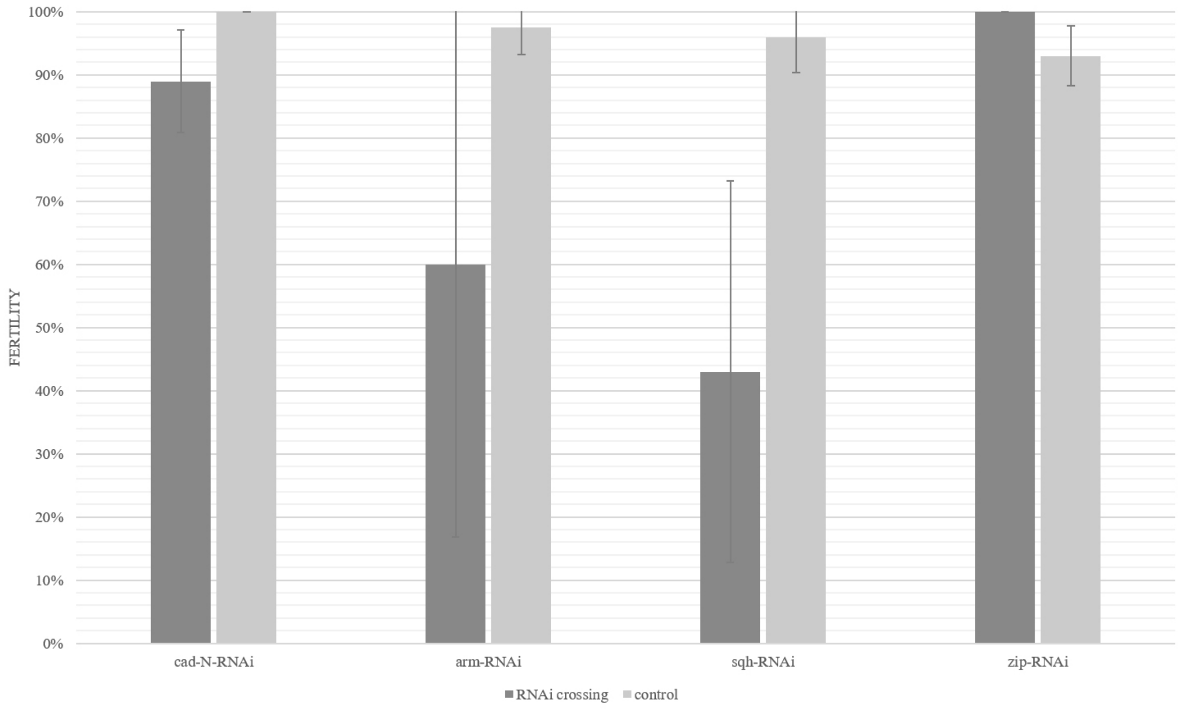

Figure S1: Fertility of males after RNAi knock-down experiments. Mef2- or Mef2>>Dcr2-driven RNAi males were crossed with three wild type virgin females. For controls, RNAi males were tested under the same circumstances. Neither *cad-N* knock-down nor *zip* knock-down resulted in reduced male fertility. Fertility of the males was reduced only upon down-regulation of *sqh* and *arm*. n = 30.

Biology Open • Supplementary information

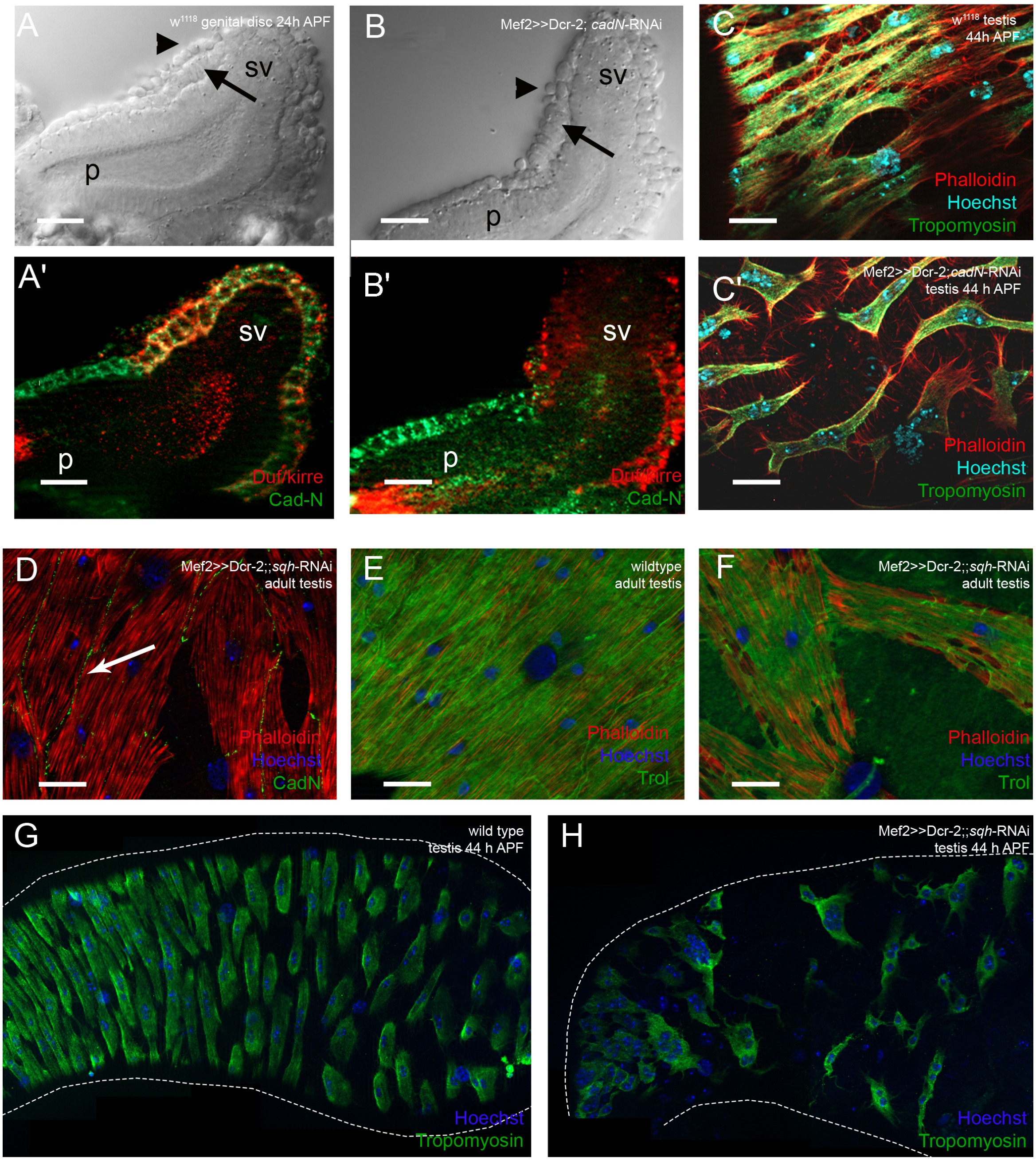

Figure S2: Reduction of Cad-N or Spaghetti squash does not influence expression of Trol in adult testes but disturbes efficient population of the testes with muscles. (A, B) Differential interference contrast (DIC) micrograph of testis 44 h APF; 8A´-H: immunolfuoresence; Phalloidin (red) labels F-actin, and Hoechst (blue) visualises nuclei. (A) On genital discs of wild type males FC-like (arrow) and FCM-like (arrowhead) myoblasts are visible in interference contrast. (A') The adhesion protein Duf/Kirre was expressed in FC-like

myoblasts on seminal vesicles 24 h APF. B) After knock down of *cad-N*, FC-like (arrow) and FCM-like myoblasts (arrow head) were detectable and B') Duf/Kirre expression was maintained. (C, C') At 44 h APF, pupal *cad-N* knock-down testes (C`) contain nascent myotubes with less elongated shapes; the number of myotubes might be reduced (compare to C, wild type). (D) Cad-N was expressed between adjacent myotubes (arrow) in *sqh* knock-down testes. (E) The ECM protein Trol was expressed in the muscle sheath of wild type testes and (F) in the irregular-shaped muscles upon *sqh* knock-down. (G) Pupal wild type testes at 44 h APF. (G) Pupal *sqh* knock-down testes at 44 h APF showed little defects in shape (compare to F), but are covered with fewer nascent myotubes. Scale bars, 20 µm.

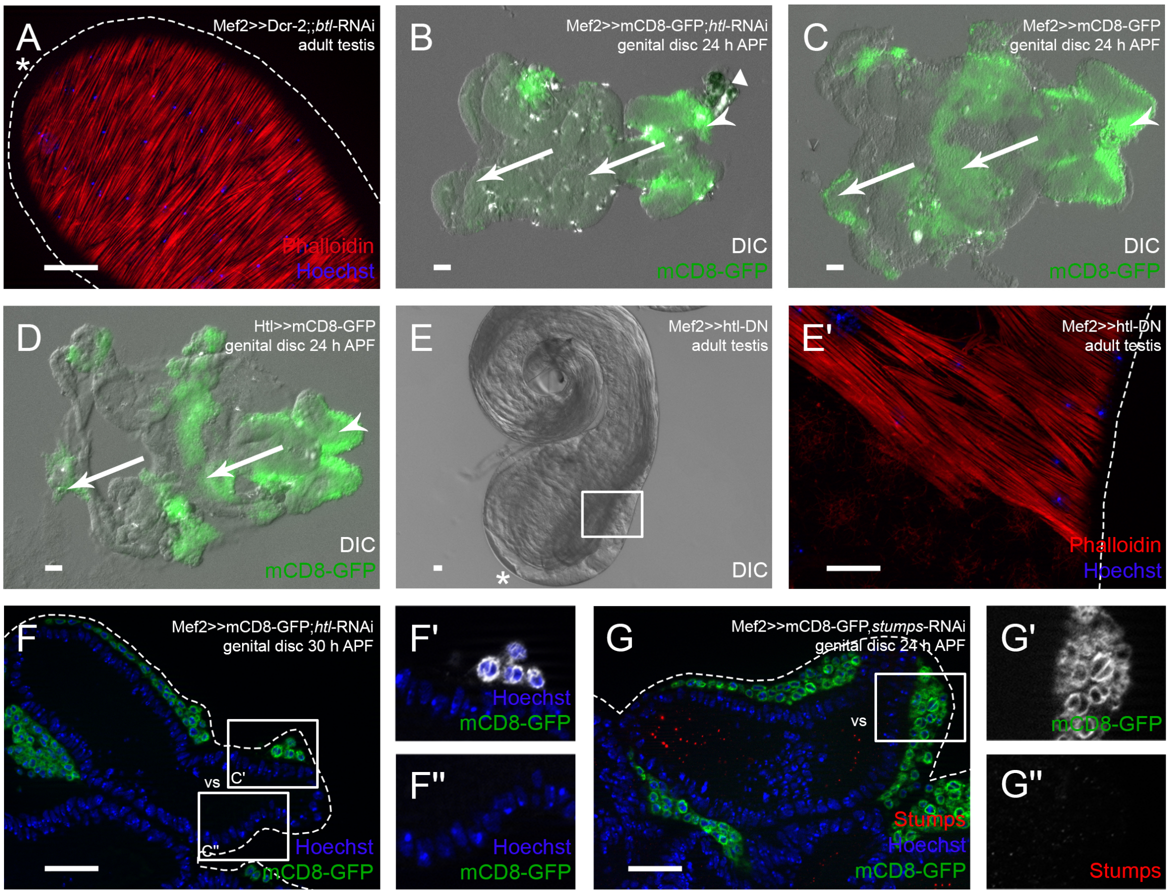

Figure S3: Analysis of FGF-signaling components (A) Down-regulation of *htl* did not affect the adult testis muscle sheath. (B) Upon *htl* knock-down, the number of myoblasts on genital discs 24 h APF was severely reduced in the posterior part (compare to C; arrows), whereas testis-relevant myoblasts accumulated over the prospective seminal vesicle. Triangle indicates fat tissue. (C) Myoblasts on genital discs 24 h APF. (D) Htl-Gal4 drives expression in posterior myoblasts (arrows) and in testis-relevant myoblasts (arrowhead) on genital discs 24 h APF. (E) Myoblast-specific expression of Htl-dominant negative (DN) produced an adult testis with a bulky tip. (E’) In the htl-DN background, testis muscles did not cover the bulky tips. (F–F’’) At 30 h APF, *htl* knock-down (BL35024) seminal vesicles exhibited a severely reduced number of myotubes. F′ and F″ show enlargements of the respective boxed areas in F. (G–G’’) On *stumps* knock-down genital discs 24 h APF, Stumps was not expressed. Phalloidin (red) visualizes F-actin, Hoechst (blue) labels nuclei. On genital discs, myoblasts and nascent myotubes are marked with Mef2-driven mCD8-GFP. Asterisk marks testis hubs. Dotted lines reflect approximate organ shape. Scale bars, 20 µm.

Table S1: Phenotypes of RNAi fly lines

**RNAi fly line**	**Testis phenotype**	**Predicted off-targets**
Mef2>>Dcr-2;*cadN*-RNAi v1092	shape: not coiled properly<br>muscle sheath: not continuous	none
Mef2>>Dcr-2;*cadN*-RNAi v1093*	shape: not coiled properly<br>muscle sheath: not continuous	none
Mef2>>*cadN*-RNAi v101642	shape: wt<br>muscle sheath: some holes	CadN2
Mef2>>Dcr-2;;*sqh*-RNAi BL32439*	shape: small and thickly<br>muscle sheath: not encircling	none
Mef2>>Dcr-2;;*sqh*-RNAi BL33892	shape: wt<br>muscle sheath: holes	none
Mef2>>Dcr-2;;*zip*-RNAi BL36727	shape: small and thickly<br>muscle sheath: big holes	none
Mef2>>Dcr-2;*zip*-RNAi BL37480*	shape: coiled with big testis tip<br>muscle sheath: holes	none
Mef2>>*arm*-RNAi BL31304*	shape: many thickenings<br>muscle sheath: short muscles, holes	Sequoia (zink finger protein)
Mef2>>*arm*-RNAi BL35004	shape: wt<br>muscle sheath: wt	none
Mef2>>Dcr-2;;*btl*-RNAi BL40871	shape: wt<br>muscle sheath: wt	none
Mef2>>Dcr-2;*btl*-RNAi v27106*	shape: wt<br>muscle sheath: wt	none
Mef2>>Dcr-2;;*htl*-RNAi BL35024	shape: coiled with big testis tip<br>muscle sheath: no muscles at tip	none
Mef2>>*htl*-RNAi v6692*	shape: small and round, no connection to vs<br>muscle sheath: no muscles	none
Mef2>>*stumps*-RNAi v21317*	shape: variable, small and round, but connected to vs<br>muscle sheath: variable, no muscles at apical region	none

Mef2>>*stumps*-RNAi v105603	shape: variable, small and round, but connected to vs<br>muscle sheath: variable, big regions without muscles	none

wt = wild type; vs = seminal vesicle; * = presented in Results

Biology Open • Supplementary information
